# Hemostasis Strategies and Recent Advances in Nanomaterials for Hemostasis

**DOI:** 10.3390/molecules28135264

**Published:** 2023-07-07

**Authors:** Jian Du, Jingzhong Wang, Tao Xu, Hai Yao, Lili Yu, Da Huang

**Affiliations:** 1 Suining Municipal Hospital of Traditional Chinese Medicine, Suining 629000, China; dujian19802004@163.com (J.D.); wjz19870821@126.com (J.W.); woxutao125@163.com (T.X.); 2 Center For Peak of Excellence on Biological Science and Food Engineering, National University of Singapore (Suzhou) Research Institute, Suzhou 215004, China; hai.yao@nusri.cn; 3 College of Biological Science and Engineering, Fuzhou University, Fuzhou 350108, China

**Keywords:** effective hemostasis, hemostasis strategies, nanomaterials, wound healing, nanotechnology

## Abstract

The development of materials that effectively stop bleeding and prevent wound adhesion is essential in both military and medical fields. However, traditional hemostasis methods, such as cautery, tourniquets, and gauze, have limitations. In recent years, new nanomaterials have gained popularity in medical and health fields due to their unique microstructural advantages. Compared to traditional materials, nanomaterials offer better adhesion, versatility, and improved bioavailability of traditional medicines. Nanomaterials also possess advantages such as a high degree and stability, self-degradation, fewer side effects, and improved wound healing, which make them ideal for the development of new hemostatic materials. Our review provides an overview of the currently used hemostatic strategies and materials, followed by a review of the cutting-edge nanomaterials for hemostasis, including nanoparticles and nanocomposite hydrogels. The paper also briefly describes the challenges faced by the application of nanomaterials for hemostasis and the prospects for their future development.

## 1. Introduction

Uncontrolled wound bleeding (war, accident, etc.) and surgical bleeding are the main causes of death [1,2,3]. Excessive bleeding will prolong the operation time and increase the risk of surgery and even lead to medical malpractice. Effective control of blood loss is able to save time and improve the survival rate of patients [4]. When bleeding occurs, hemostasis is the body’s spontaneous response. The mechanism of hemostasis in vivo involves two processes: primary hemostasis, when endothelium gets injured, and collagen and other subendothelial matrix components are exposed, and von Willebrand factor is released to allow platelets to adhere to the wound site; secondary hemostasis, tissue factor stimulates the conversion of prothrombin to thrombin, and soluble fibrinogen acts to limit the formation of insoluble fibrin clots [5]. However, in the event of massive bleeding, the body cannot rely on the natural hemostasis process to control blood loss, and an effective external hemostasis method is required to control the body’s blood loss. Therefore, additional methods are needed to stop bleeding quickly during medical assistance [6].

The clinical goal is to combine hemostasis technology at the trauma site to effectively prevent internal or external bleeding. However, in emergencies, there are many problems with traditional hemostasis methods, such as inconvenient use, short validity period, and unsatisfactory hemostasis effect. Chemical hemostatic agents (chitosan-based products, biologically active agents, antifibrinolytics), are more commonly used than mechanical hemostasis and thermal hemostasis, and have outstanding advantages: easy to carry, easy to use, and can effectively stop bleeding in emergency situations [7].

Therefore, it is still an urgent problem to develop new hemostatic materials to effectively control bleeding and strive for the survival rate of severely injured patients. Recently, nanomedicine has been utilized to improve the efficacy of different hemostatic agents in the form of nanoparticles, nanofibers, or nanocomposites [8]. Compared with traditional hemostatic materials, nanomaterials can be modified in microstructure, perform good diffusion and solubility, can better adhere to wounds and release drugs for targeted drug delivery, and improve the biological properties of traditional hemostatic drugs. Furthermore, nanomaterials are able to improve the stability of hemostasis [9]. With the improvement of medical technology, various nanomaterials have been used to stop bleeding and prevent wound adhesion. This paper firstly briefly summarizes the current hemostatic methods and materials, and subsequently introduces the current research status of nanomaterial technology according to classification. After that, the advantages of nanomaterials in stopping bleeding and preventing wound adhesion have been introduced. Finally, the problems and development prospects of nano hemostatic materials are put forward.

## 2. Physiological Hemostatic Mechanisms

The process of physiological hemostasis in the body includes three steps: the first is vasoconstriction, that is, the integrity of the vascular walls and vascular endothelial cells are damaged, and the subendothelial matrix is exposed to the blood, which causes platelet capture, adhesion, and activation. Finally, a platelet plug is formed. At this time, the smooth muscle in the blood vessel contracts to prevent blood loss at the damaged part of the tissue. The smooth muscle contracts in the larger blood vessel to effectively stop bleeding, and reduces further damage to the wound [10]. The second step is primary hemostasis, which is mainly related to platelets and involves platelet activation, adhesion, secretion, aggregation, and contraction. When the body is not injured, the continuous production of prostacyclin and antithrombotic nitric oxide in the body prevents platelets from adhering to the blood vessel wall. When the body is injured, the wound exposes the subendothelial matrix, and primary hemostasis initiates. Firstly, the coagulation cascade was further activated through von Willebrand factor (VWF) thrombogenic components such as collagen and procoagulant protein, and platelets are further activated by collagen, adrenaline, adenosine diphosphate, and thrombin by binding with these components. After activation, platelets change their shape to further enhance their adhesion. Activated platelets achieve hemostasis by releasing mediators that attract more platelets to form thrombosis. The temporary clot, driven by platelets, is present only at the wound site, thereby reducing complications from thromboembolism. The last is secondary hemostasis, which activates the coagulation cascade and forms a powerful blood clot through fibrin deposition on the wound, completely stopping the blood loss of the wound. When blood vessels rupture, factor VIIa and its cofactor tissue factor (TF) in extravascular tissues form a complex to activate the reaction dominated by coagulation cascade serine protease. The complex will activate factor IX and factor X at the same time. The activation of coagulation is initiated by the reaction of activating factors IX and X. Once a critical amount of factor Xa is produced, thrombin production begins. Thrombin regulates the conversion of fibrin from soluble to insoluble. Fibrin formation occurs simultaneously with platelet aggregation, and both of them attach to the wound. Thrombin also activates factor XIII, which directly enhances the formation of blood clots by controlling the crosslinking of fibrin webs [7,10].

**Figure 1:**
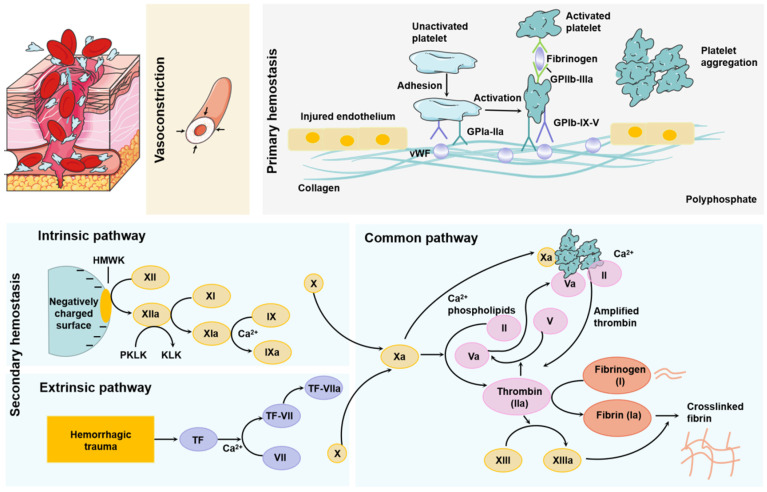
Schematic of the complex mechanism of blood vessel hemostasis.

There are two ways to promote blood coagulation, including the tissue factor pathway (TF pathway) and the contact pathway. These two pathways activate the coagulation cascade. Tissue factor pathway: cell surface complex activates FIX and/or FX through limited proteolysis. Contact route: FXII, PK, and HK are assembled, which leads to FXII being mutually activated by kallikrein to FXIIa, while PK is activated by FXIIa to kallikrein. The generated FXIIa activates FXI to FXIA, and then converts FIX to FIXA. Ultimately, the common pathway promotes the production of thrombin, which activates platelets [11]. The mechanism of hemostasis is shown in Figure 1.

## 3. Physical Hemostatic Methods

Normally, hemostasis is achieved when adherent platelets and coagulation factors are locally abundant. However, massive hemorrhage after trauma requires manual intervention to promote blood coagulation; otherwise, it may lead to serious complications and even life-threatening. Physical hemostasis (Table 1) is widely used in extracorporeal hemostasis because of its convenience. The method of hemostasis of trauma should vary according to the type of vascular bleeding (traumatic and internal bleeding). The amount of capillary bleeding is small, with slow blood flow. This can be wrapped with medicine and gauze after washing with clean water. The treatment of arterial bleeding is usually to stop bleeding with finger pressure near the heart or with a tourniquet to stop bleeding quickly. The method of stopping venous bleeding is to hold the distal end to stop bleeding. Finger pressure hemostasis refers to the method of pressing the artery on the bone with the finger or palm on the upper end of the injured part to prevent blood circulation. This method is applicable for the treatment of arterial bleeding, which is easy to compress in the superficial part. Moreover, the hemostasis method of bending the limb with a pad is applicable to the bleeding of non-fracture trauma of the limbs [12]. Another common way to stop bleeding is to apply pressure on a blood vessel with a rubber tube. The method is reliable, but only applicable to limb arterial bleeding. Excessive wrapping of the tourniquet will lead to skin damage, and prolonged tourniquet entangling can promote tissue ischemia and necrosis.

Pneumatic hemostasis fills the tourniquet with gas through a high-efficiency air pump, which can compress the limbs, block the blood flow and achieve the effect of hemostasis [13]. This method is generally used in orthopedic surgery to prevent wound bleeding to the greatest extent, make the surgical vision clear and avoid minor structural damage, while it will cause paralysis, shock, pain, skin damage, tissue ischemia, and other adverse reactions.

The surgical hemostasis methods that can be used for internal bleeding include single ligation hemostasis, hemostasis through hemostatic forceps, and the ligated tissue is completely sheathed by a ligation line, which may cause postoperative bleeding due to inaccurate ligation or falling off, and too tight will cut blood vessels. In suture ligation hemostasis, through ligation, the ligation line can be avoided from falling off [14]. The suture takes a long time, the suture consumes a lot, and the tissue may not be aligned. In animal models, tying sutures is often used for hemostasis. Electrocoagulation hemostasis is to use the probe to contact the vicinity of the wound. The coagulation current will produce high heat, promote the edema of the tissue around the wound, reduce or block the compressed vascular cavity, and form thrombus hemostasis. This method is simple, safe, and economical. The disadvantage is that too long electrocoagulation time will lead to larger and deeper trauma, resulting in bleeding again [15].

Ultrasonic cutting and blood-stopping knife is a new type of ultrasonic treatment equipment used in open and endoscopic surgery. Its working principle is to amplify the kinetic energy on the knife rod and cut the tissue through the ultrasonic system in the handle. After the tissue in contact with the knife head absorbs the ultrasonic energy, the protein hydrogen bond breaks, then solidifies and denatures, and is cut under the clamping pressure of the jaw, so as to achieve the effect of cutting and coagulation. At the same time, the water in the tissue vaporizes to further help tissue stratification. Ultrasonic scalpel hemostasis has many advantages: a wide range of use, clear field of vision during operation, fast cutting, small cutting tissue damage, etc., but it also has the disadvantages of slow operation, high price, limited cutting range and so on [16].

Laser hemostasis is the use of laser coagulation hemostasis, the use of heat energy to evaporate the water in cells, promote the degeneration and contraction of vascular wall collagen, and form small vascular thrombosis, and the pressure and impact of the laser also play a role in hemostasis [17]. It has little damage to surrounding tissues, is effective in hemostasis of capillaries and arterioles, and sterilizes at the same time. However, it will produce toxic smoke and is easy to adhere to after operation [18].

Microwave knife hemostasis generates heat energy through the radiation of the microwave knife head, and the tissue absorbing heat energy will solidify. After resection, it can achieve a hemostasis effect. The hemostasis effect is obvious; it is not easy to produce a burning taste, the wound heals quickly, and there is less postoperative bleeding, but it only solidifies the blood vessels within 3 mm and temporarily closes the blood vessels [19].

The principle of radiofrequency knife hemostasis is to form a small plasma electric field in the electrolyte through radiofrequency energy. After the acceleration energy is sufficient, the energy is transmitted to the tissue; the protein ion bond is destroyed and coagulated. The thermal effect is small, and the damage is small. When hemostasis is stopped, saline drops at the same time. The hemostasis effect is good. It can only coagulate blood vessels below 2 mm. A large amount of normal saline is required in the liquid environment. The operation is inconvenient due to the brine path [20].

Argon knife hemostasis can conduct high-frequency current to the tissue through an ionized gas, and the thermal effect can play a good therapeutic effect. The utility model has the advantages of stable gas, harmless inert gas, no smoke during operation, and a clear field of vision. Tissue damage is small, the depth is less than 3 mm, does not contact the wound, continuous solidification, small thermal effect, dense eschar can be formed, and the hemostatic effect is better. The disadvantage is that it can only coagulate blood vessels < 2 mm, which may increase pneumoperitoneum pressure and promote gas embolism and vascular gas embolism [21].

Ablation hemostasis: an ablation electrode is a hemostatic device in surgery, which is generally used together with various RF manipulators. Its principle is mainly to use the thermal tissue effect to dehydrate the tissue and further lead to protein degeneration, coagulation, and necrosis. Electrocoagulation and electrocision are carried out on the tissue in surgery, so as to stop bleeding and cutting. Ablation electrode has many advantages, ≤5 μM needle tip is convenient for minimally invasive surgery. The shorter needle tip can effectively avoid accidental tissue injury and reduce tissue adhesion. It can operate under low power, reduce tissue injury and scar, shorten postoperative healing time, and is not easy to produce harmful smoke and clear vision. However, the tissue temperature during RF ablation depends on the power and discharge time. If the tissue temperature exceeds 50 degrees, it is easy to cause damage. The higher the tissue temperature is, the deeper the damage is. Heating more than 100 degrees will produce bubbles and cause perforation. Under the power control mode, it will lead to tissue overheating and poor gasification safety. Under the temperature control mode, it may be due to the slow blood flow rate of patients; Scab makes the temperature of the electrode tip high and prolongs the discharge time, resulting in low operation efficiency [22].

Although these surgical hemostatic measures can temporarily organize bleeding, hemostatic accessories are still needed for adjuvant treatment. Antifibrinolytic drugs can inhibit the disintegration of blood clots and improve the sealing effect of wounds. However, the reduction of the activity of the fibrinolytic system in vivo may promote the formation of thrombosis and bring side effects of treatment. Therefore, it is urgent to study and apply some new nanomaterials that can promote wound healing, effectively stop bleeding, and prevent adhesion [23,24,25,26].

## 4. Commonly Used Hemostatic Materials

Traditional hemostatic materials, such as tourniquets, sponges, and gauze, have a good effect on superficial hemostasis of the skin, which can prevent blood outflow in a short time and reduce further deterioration [27,28]. However, traditional hemostatic methods and materials have limitations in internal bleeding or compressive important organs such as the brain, liver, etc. For example, if gauze or bandage is not degradable, the long-term wrapping may delay healing and produce secondary injury and additional pain [29]. Degradable hemostatic materials have been explored (Table 2). Compared with the traditional hemostasis methods, the injectable and fluidity of hydrogels make them more effective in hemostasis in vivo and promote wound healing in tissues and organs in the body. Its biocompatibility ensures the safety of hydrogel materials. These biomaterials can combine additional functions as needed, such as material-carrying cells or drugs. However, its clinical application is still limited by the disadvantages of uncontrollable degradation and weak mechanical ability [30]. Compared with these shortcomings, synthetic materials can be modified to obtain the desired properties, so as to better adhere to the damaged tissue for hemostasis. At the same time, it is also necessary to consider whether the toxicity of synthetic materials has an adverse impact on the body itself. In addition, intravenous injection of antifibrinolytic drugs, platelets, and platelet substitutes can regulate the balance of fibrin in the body, which can also improve the hemostatic effect (Table 2) [31].

## 5. Research and Application of Hemostatic Nanomaterials

The second leading cause of death from trauma is traumatic hemorrhage. In the pre-hospital hemostasis stage, the traditional hemostasis methods and materials have many shortcomings, such as gauze or bandage leading to secondary injury in the longer term, the low mechanical strength of natural biomaterials, the toxicity of synthetic hemostasis materials, etc. Exploring and studying new materials that can effectively control external trauma and internal bleeding has broad prospects. Currently, commonly used hemostatic materials include Surgicel, QuikClot, Celox^®^s, MPH, and Singclean’s Quickclean and Surgiclean, which all use nanomaterial technology. The Surgicel series has been widely used in surgical procedures. Although Surgicel products are generally effective, bioabsorbable hemostatic materials with proven safety and efficacy, and bactericidal properties. However, there are some safety concerns when applied to clinical treatment, including delayed absorption, umbilical cord compression, and granuloma/tumor. QuikClot, the first commercially available zeolite hemostatic, is a granular preparation that can be poured directly onto the bleeding site. It absorbs water quickly to stop bleeding, and produces an exothermic reaction, resulting in adverse side effects when applied in vivo. To avoid this side effect, second-generation QuikClot ACS has been produced, which is reported to be more effective than QuikClot, especially when applied to irregular injury sites that do not distribute freely into the wound. The product is also easier to remove and produces less exothermic reactions than QuikClot. Celox^®^s is a chitosan-based hemostatic product for patients with coagulation dysfunction. Hemostatic gauze made of chitosan, silica, and polyethylene has a larger specific surface area, which can significantly increase the contact area with the wound. MPH is a hydrophilic plant polysaccharide hemostatic that accelerates the clotting process by absorbing water from the blood, concentrating platelets, and clotting proteins. MPH has been reported to reduce seroma after mastectomy in rats [32].

Nanotechnology can transform and utilize the microstructure on the nanoscale, which gives nanomaterials unique advantages such as improved diffusivity and solubility, easy-to-penetrate physiological barriers, large specific surface area, slow control, and targeted release of drugs. For example, during primary hemostasis, platelets can be activated to stop bleeding [33]. One of the mechanisms by which nanomaterials can stop bleeding is by activating platelets by mimicking platelet-activating factors. Some researchers used this hemostasis mechanism to prepare collagen mimetic peptides formed by nanofibers to activate platelets and thus stop bleeding [34]. In addition, the parameters of nanomaterials, such as roughness, are correlated with platelet activation [35]. Secondary hemostasis has two enzymatic pathways: intrinsic and extrinsic pathways. The intrinsic pathway is activated by a negative charge on the connective tissue, leading to the activation of factors XII as well as XI and IX [36,37]. Thus, negatively charged nanomaterials can activate factor XII and initiate intrinsic pathways. The in vitro pathway is triggered by tissue factors released from tissue damage [38,39], and nanomaterials with cations can stop bleeding by activating the in vitro pathway [40]. In recent years, researchers have made more and more in-depth research on nano-hemostatic materials such as nanosheets, liposomes, nanoparticles, and self-assembling nano-peptides, which provide more reference for the development of hemostatic materials.

### 5.1. Nanoparticles

**Figure 2:**
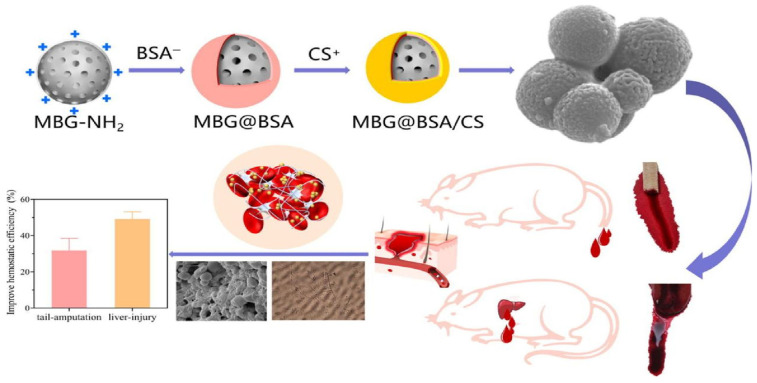
Hemostatic mechanisms of bioactive glass composite particles [45]. Copyright © 2022 The Author(s). Published by Elsevier Ltd.

Nanoparticles refer to colloidal particles composed of macromolecular substances with a solid particle size of 10–1000 nm. Charged nanoparticles can produce an electrostatic effect with blood cells or fibrinogen with opposite charge, neutralize the surface charge, induce its aggregation, and promote blood coagulation [41]. Blood is an important medium to help nanoparticles reach target tissues and organs. Moreover, they also have unique flow influence characteristics, which can affect the distribution of platelets and nanoparticles in the vascular system [42,43]. Additionally, nanoparticles can easily penetrate biological barriers, enter systemic circulation, and penetrate cells via endocytic processes, including pinocytosis, phagocytosis, or endocytosis [44]. Nanoparticles cause changes in erythrocytes, which affect the viscosity of blood and thus play the role of hemostasis. Zheng et al. encapsulated bovine serum albumin (BSA) and chitosan (CS) in a mesoporous bioactive glass (MBG) nanoparticle (MBG@BSA/CS) that activates physiological coagulation pathways. Significant effects were observed on both surface and internal bleeding in SD rats (see Figure 2 below) [45]. Blood compatibility is one of the main standards for approving all nanomedical devices in contact with blood. Silver nanoparticles (AgNPs) with antibacterial properties and the potential to promote platelet aggregation are one of the more commonly used hemostatic nanomedical materials, which can cause dose-dependent hemolysis. Researchers used sodium bis (2-ethylhexyl) sulfosuccinate (AOT), polyvinylpyrrolidone (PVP) Silver nanoparticles coated with polylysine (PLL) and bovine serum albumin (BSA) delayed plasma coagulation. Only PLL-type AgNPs could inhibit plasma coagulation and induce platelet activation, thereby interfering with hemostasis [46]. Red blood cells and platelets are key participants in the coagulation process. They promote coagulation through the procoagulant activity (PCA) complex expressed on their surface. Blood coagulation is a complex process that involves not only these cells in the blood but also biochemical components such as coagulation factors. The coordinated interaction of these key cells and biochemical components is necessary to maintain hemostasis and prevent excessive bleeding. Causing platelet aggregation, interfering with plasma coagulation, and inducing the production of leukocytes, PCA can be used to measure the possibility of nanomaterials promoting coagulation or anticoagulation in vivo and in vitro. In vitro test shows that the adjustment of nanoparticle concentration can effectively regulate platelet aggregation, interfere with plasma coagulation and activate leukocyte PCA [47]. However, most studies focus on the function of nanoparticles and ignore the potential risk that nanoparticles may disrupt the hemostatic balance and may also lead to thrombosis and bleeding [48].

### 5.2. Nanosheets

**Figure 3:**
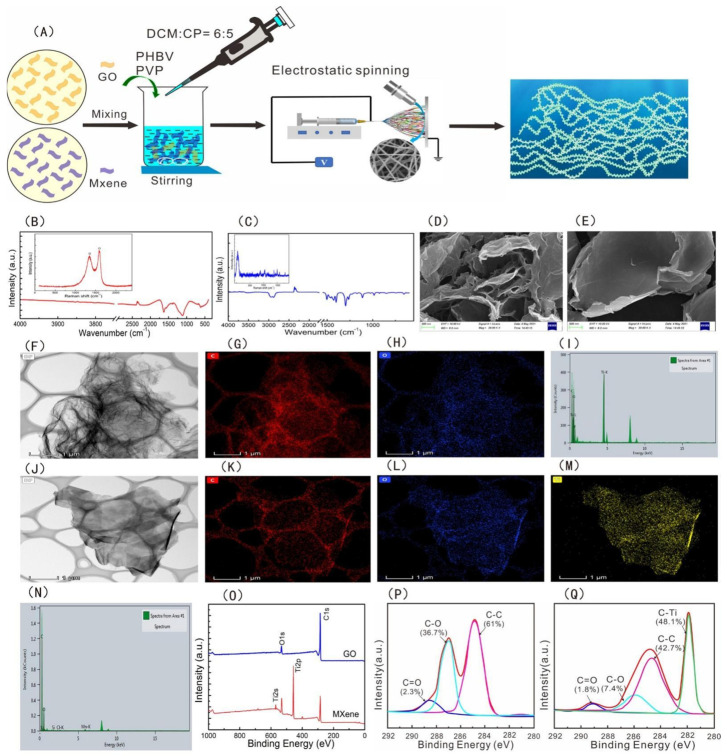
(**A**) Schematic diagram of preparation of the PHBV−GO/MXene composite membranes. FT−IR and Raman spectra of (**B**) GO and (**C**) MXene. SEM images of (**D**) GO and (**E**) MXene. TEM analysis of (**F**) GO and (**J**) MXene. Distribution of elements: (**G**) C for GO, (**H**) O for GO, (**K**) C for MXene, (**L**) O for MXene, and (**M**) Ti for MXene. The element composition of (**I**) GO and (**N**) MXene. (**O**) XPS spectra of GO and MXene. C1s XPS spectra of (**P**) GO and (**Q**) MXene [49]. Copyright © 2021 by the authors. Licensee MDPI, Basel, Switzerland.

Freestanding super thin film, often referred to as nanofilm or nanosheet, is a new two-dimensional nanomaterial that has attracted much attention in the field of nanotechnology in recent years. Nanosheets have a large surface area/aspect ratio with high transparency and superior flexibility. When applied to wounds, platelets, blood cells, and coagulation factors can be concentrated through rapid water absorption. The negative charge on its surface can activate the endogenous coagulation pathway, so as to achieve the purpose of hemostasis. For example, Wu et al. prepared a novel nanofilm with high antibacterial hemostatic ability by embedding GO/MXene nanosheets uniformly on the surface of PHBV fibers. Compared with the PHBV membrane, the tensile strength, platelet adsorption, and clotting time of the membrane with nanosheets were significantly increased, and the antibacterial rate of the membrane could reach 97% (see Figure 3 below) [49]. Shi et al. combined montmorillonite nanosheets with Lycium barbarum polysaccharide to prepare hemostatic hydrogel, which had a three-dimensional porous structure and hydrophilic surface, which was conducive to the rapid adsorption of blood. In addition, in vivo experiments have proved that it can effectively reduce the tissue damage caused by inflammation and shorten the wound healing time, which has great application potential in the clinical treatment of hemostasis and wound healing [50]. Xuan et al. developed a GDP@Ca^2+^/PCL nanosheet, which has high strength, can stop bleeding and be antibacterial, and can adhere to uneven tissues in vivo environments. In addition, by combining Ca^2+^ and antimicrobial peptides, the nanosheet demonstrated excellent hemostatic and anti-infective properties in mouse models of back skin and liver damage [51]. However, the mechanism of the nanosheet on the coagulation process is not clear, and the interaction between the material and platelets and blood cells, as well as the stimulation of the endogenous coagulation pathway, need to be further studied.

### 5.3. Liposomes

**Figure 4:**
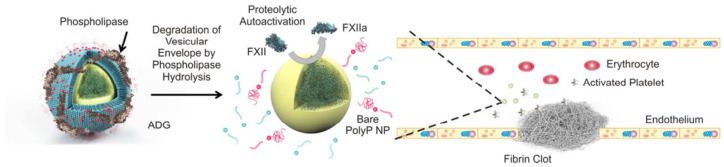
The preparation and hemostatic mechanism of procoagulant liposome [56]. Copyright © American Chemical Society.

Liposomes usually refer to spherical bilayer lipid molecules with a diameter of 20 nm to 10 μm made of phospholipids and cholesterol. The properties, types, surface charges, particle sizes, and preparation methods of liposomes vary widely. It is a widely studied nano-delivery system [52]. Liposomes can encapsulate coagulation factors, prevent coagulation factors from being cleared and inactivated, and improve the circulation time of coagulation factors in vivo. Liposomes can also be coupled with the hemostatic polypeptide chain to enhance the stability of the polypeptide chain while exerting its hemostatic effect [53]. Modery-Pawlowski et al. modified liposomes with collagen and VWF-binding peptides, CBP and VBP, to promote platelet aggregation. This liposome construct has shown significant hemostatic effects in a mouse tail amputation model [54]. In addition, researchers enclose thrombin in liposomes and deliver it to isolated platelets, which will allow transfusion platelets to coagulate more easily in response to platelet agonists and eliminate the increased risk of thrombosis caused by thrombin delivery [55]. Furthermore, nanoparticles have been applied to deliver components that activate coagulation factors, and combined liposomes with nanoparticles to prepare new hemostatic nanomaterials. For example, Donovan et al. encapsulated polyphosphate nanoparticles into liposomes, and this nanomedicine can shorten the clotting time of plasma and show good procoagulant properties (see Figure 4 below) [56].

### 5.4. Nanofibers

**Figure 5:**
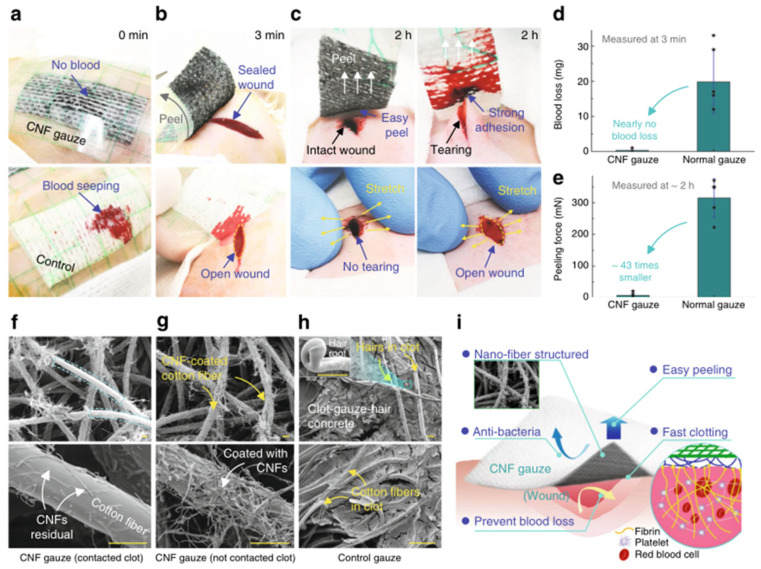
In vivo animal experiment on hemostasis by nanofibers. (**a**) The plaster-like gauzes were patched onto incisions on rat back; the control cotton gauze got wet quickly, while the CNF gauze prevented blood loss. (**b**) Peeling the gauze at 3 min to measure the blood loss; the CNF gauze helped form a gel-like clot, which properly sealed the wound; under the control gauze, an open wound was observed. (**c**) Peeling the gauze at about 2 h to measure the peeling force; the CNF gauze could be easily peeled off and gently stretching the wound did not cause wound tearing or bleeding); in contrast, peeling the normal gauze caused wound tearing and bleeding. (**d**) The CNF gauze minimized blood loss (**n** = 6). (**e**) Peeling force for the CNF gauze was significantly smaller than that for the normal gauze (**n** = 5). (**f**) SEM images of the area in contact with blood on the CNF gauze in c; CNF residuals after clot detachment were observed on cotton fibers. (**g**) SEM images of the area not in contact with blood on the CNF gauze in c, where cotton fibers were densely coated with CNFs. (**h**) SEM images of the peeled normal gauze in c, showing a clot-gauze-hair concrete, with rat skin hairs imbedded in clot; a hair root is shown in the inset, implying that skin hairs that were stuck in the clot were pulled out from skin during gauze peeling. (**i**) Schematic of the hemostatic CNF gauze/plaster for wound treatment. Data in d and e are shown as mean ± SD, the error bar represents SD, and individual data points in (**d**,**e**) are represented by * [29]. Copyright © 2019 Li, Milionis, Zheng, Yee, Codispoti, Tan, Poulikakos and Yap.

Nanofiber refers to a linear material with a certain length diameter ratio with a diameter of nanoscale and a large length. In a narrow sense, the diameter of nanofiber is between 1 nm and 1000 nm. Nanofiber has the characteristics of surface effect, easy combination with other atoms, small size effect, such as melting point reduction, color separation, and discoloration, quantum size effect, macro quantum positive tunnel effect, etc. Nanofibers can mimic the nanomorphology characteristics of fibrin fibers during natural hemostasis, and have a large surface area, adjustable porous structure, and precisely controllable structure; hence nanofibers have a strong potential for hemostasis (see Figure 5 below) [57,58]. Xianrui Xie and others prepared an ultra-light 3D gelatin sponge composed of nanofibers. In vitro evaluation showed that the sponge had good cell compatibility, high cell permeability, and low hemolysis rate. Subcutaneous implantation studies in rats have shown that the sponge aggregates and activates large numbers of platelets accelerates embolism formation, and promotes other coagulation pathways to accelerate clotting. The in vivo study of the rabbit ear artery injury model and liver injury model shows that compared with commercial gelatin hemostatic sponge, gelatin nanofiber sponge can quickly induce stable thrombosis and perform the least blood loss. The above findings suggest that gelatin nanofiber sponge is a potential absorbable hemostatic agent [59]. Shixuan Chen et al. have developed an injectable and super elastic nanofiber matrix. Compared with commercialized hemostasis materials, the matrix exhibits greater absorption/blood capacity and has high efficiency in whole blood coagulation assay, especially for thrombin-immobilized samples. Further in vivo tests showed that the nanofiber matrix was effective in hemostasis in the pig liver injury model [60]. Li et al. used carbon nanofibers (CNFs) to promote fibrin growth and cause rapid clotting, with super fiber hydrophobicity limiting blood wetting to prevent blood loss and antibacterial. In addition, after the clot shrinks, it can be peeled off automatically, avoiding tissue damage. The above characteristics of the material make it a good application in the field of hemostasis [29]. Other researchers expanded the two-dimensional (2D) nanofiber membrane to three-dimensional space to obtain a three-dimensional (3D) layered nanofiber sponge, which increased the interface interaction between sponge and blood cells and accelerated hemostasis. The structural adjustment of this nanofiber shows good elasticity, high permeability and liquid absorption rate, and high compressibility and elasticity, which is conducive to filling the deep wound and promoting tissue healing. Moreover, the 3D dynamic environment can regulate the tissue cells of the wound and promote the regeneration of the dermis and the recovery of cells. Full-thickness skin defect experiments in mice showed that 3D layered nanofibers effectively accelerated wound healing and reduced scar formation [61].

### 5.5. Self-Assembling Peptides

**Figure 6:**
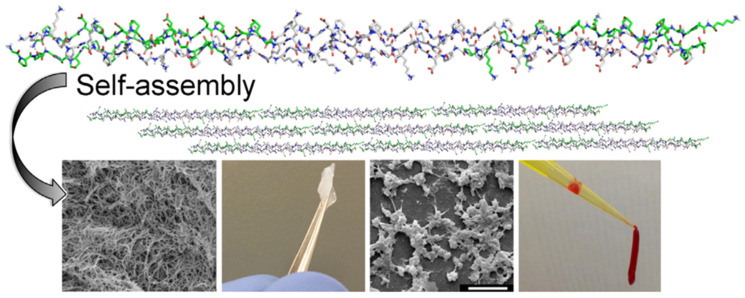
Synthetic collagen membranes consisting of Self-assembly collagen mimetic peptides are used for hemostasis [34]. Copyright © 2014 American Chemical Society.

The ordered nanostructured peptides formed by relatively simple peptide chains through noncovalent self-assembling nanopeptides are able to form a nanofiber barrier in any humid ionic environment in the body and concentrate blood components to control bleeding. It can be decomposed into natural amino acids in vivo with good biocompatibility. Min Wu et al. synthesized a hemostatic agent by solid-phase synthesis, which is a bifunctional, biodegradable, self-assembling nanopeptide (SAP) ADA16-I. The (SAP) ADA16-I solution will self-assemble into a barrier to prevent blood flow and promote the movement of adjacent cells to repair the damaged part. In the mouse model of the brain, femoral artery, and liver incision, local treatment with different concentrations of (SAP) rada16-iI solution can significantly shorten the hemostatic time. At the same time, it has the ability to bone repair. When applied to the New Zealand rabbits, it was found that the bone wax in the control group inhibited osteogenesis, while ADA16-I showed effective bone regeneration function in radiological analysis and histological examination with no serious inflammatory reaction [62]. Kumar et al. assembled a collagen-mimetic peptide (KOD) to form spiral nanofibers that promote thrombosis. KOD can activate platelets and coagulate plasma and blood. In addition to promoting thrombosis, it can also promote the production of pro-inflammatory factors (TNF-α or IL-1β). This novel self-assembling collagen mimetic peptide has good hemostatic properties (see Figure 6 below) [34]. Another research applies the peptide amphiphilic PA self-assembling into nanofibers as the carrier for delivering targeted tissue factor (TF), and chose tissue factor as the target because it was only exposed to the intravascular space when the blood vessel was ruptured and delivered to the damaged site. In the mouse great saphenous vein laser injury model, the self-assembling nanofibers reduced the blood loss by 35% to 59%; TF-targeted nanofibers can selectively locate the injury and TF exposure sites while reducing blood loss [63]. Another study developed an intravenous targeted tissue factor (TF) nano-therapy to stop bleeding. Three tissue factor-specific binding peptides were covalently bound to the backbone of the PA peptide chain and self-assembling into three kinds of nanopeptide fibers. All nanofibers were able to bind to liver bleeding sites, but only RTL nanofibers reduced blood loss by 53% compared with sham surgery, and increasing the density of targeted ligands of RTL nanofibers resulted in better binding to injury sites and reduced blood loss in vivo. Peptides successfully bound TF in vitro and successfully bound TF-targeted PA nanofibers to bleeding sites, thereby reducing blood loss in vivo [64].

### 5.6. Nanocomposite Hydrogel

**Figure 7:**
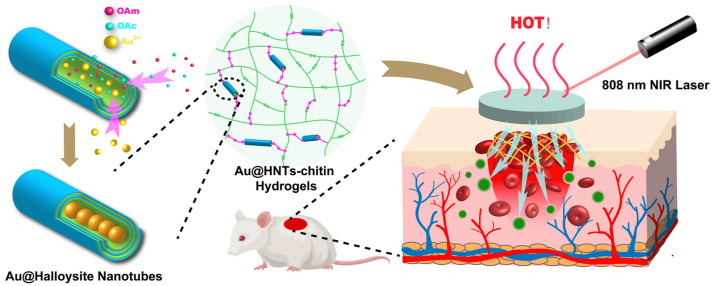
The preparation and characterization of Au@HNTs-Chitin composite hydrogel [69]. Copyright © 2022 The Authors. Publishing services by Elsevier B.V. on behalf of KeAi Communications Co., Ltd.

Gels are high molecular polymers with a three-dimensional network structure that can absorb large amounts of water or biological fluids. When the polymer structure of the gel contains hydrophilic groups, the gel will be more absorbent. The gel can swell in water without dissolving due to its three—dimensional crosslinking structure [65]. However, low mechanical strength limits the application of the gel. In recent years, nanocomposite hydrogels have become a new type of biomaterial due to the improvement of shear thinning properties [66]. The properties of hydrogels can be enhanced by adding a variety of nanomaterials as nano-fillers into the soft polymer matrix, so as to form nanocomposite hydrogels with improved properties. Nanocomposite hydrogels not only have high hydrophilicity, strong water absorption, and flexibility, but also have large specific surface area and strong adsorption capacity. Nanocomposite hydrogels can concentrate blood cells and platelets, and help to stop bleeding. At the same time, a layer of gel film is formed on the surface of the wound to block the wound. Many acute injuries result in irregular wounds or intracavitary bleeding, preventing thin films or sheets of hemostatic material from penetrating deep into the wound to being effective. Nanocomposite gels, on the other hand, come in powder form and form a gel in situ, which can be used for hemostasis in irregular and deep wounds. These physical properties make gels more similar to living tissues, so gels are gradually used as alternative materials for biological tissues [67]. Sundaram et al. combined nano-bioglass (nBG) with silica, calcium, and phosphate ions into chitosan (Ch) hydrogel as a hemostatic agent. In the analysis of the hemostatic effect in vitro and in vivo, compared with control 2% Ch hydrogel, 2% Ch- 5% nBG hydrogel formed rapid blood clots, which indicates great potential to effectively stop bleeding of nanocomposite gels [25]. Chen et al. prepared a composite hydrogel by mixing pectin and cellulose. This composite hydrogel has a dense structure while retaining the crystal structure of cellulose I and II and good thermal stability. In vivo experimental results showed that liver wounds treated with hydrogel reduced bleeding within 3 min, further highlighting the potential of the composite hydrogel as a biomedical material for rapid hemostasis [68]. Zhao et al. combined the advantages of biomacromolecules and clay to prepare a hydrogel dressing with Eloxite nanotubes and chitin as the main components. Au@HNTs-Chitin composite hydrogel has high antibacterial and hemostatic activity with low cytotoxicity, which shows the function of promoting wound healing. This research shows broad application prospects in wound antiseptic hemostasis (see Figure 7 below) [69].

## 6. Conclusions

When a large number of hemorrhagic injuries occur, the inherent hemostatic mechanism of the body is insufficient to stop bleeding in time. In order to obtain more pre-hospital first aid time for the wounded, it is necessary to adopt some techniques to achieve rapid hemostasis to improve the probability and quality of survival. Traditional hemostatic methods, such as bandage, tourniquet, gauze, and pressure hemostasis, rely mainly on physical obstruction and self-coagulation system to assist hemostasis and healing, but may face additional problems and risk of infection. Meanwhile, although internal surgical hemostatic measures can temporarily organize bleeding, hemostatic accessories are still needed for adjuvant treatment. Antifibrinolytic drugs are able to inhibit the disintegration of blood clots and improve the sealing effect of wounds. However, the reduction of the activity of the fibrinolytic system in vivo may promote the formation of thrombosis and bring side effects of treatment. Polysaccharides in commonly used hemostatic materials show the ability to promote hemostasis, accelerate wound healing, and have antibacterial properties. In contrast, the lack of adhesive strength and mechanical properties limits the application of polysaccharides in the preparation of hydrogel adhesives. Due to the high mechanical strength of protein biomaterials, protein-based hydrogels have been widely developed as effective materials for hemostasis and tissue healing. However, these natural materials contain many problems, such as degradability and mechanical strength. Although synthetic materials have effectively improved these disadvantages, there are still major problems that need to be overcome before clinical application, such as difficulty in achieving biosafety, hemostatic effectiveness, and practical feasibility at the same time. In recent years, the research on hemostatic technology has focused on nanomaterials for various clinical uses due to the unique advantages of hemostasis. The microstructure of nanomaterials can be used to modify or load drugs to achieve good hemostatic effects. However, as a new technology, its safety and reliability need to be further investigated. More experiments are needed to verify the safety and effectiveness of nanomaterials in order to provide new treatment methods for clinical practice.

## Figures and Tables

**Table 1:** Physical hemostatic methods for traumatic wounds and internal injuries.

Hemostatic Method	Principle	Advantage	Disadvantage
Tourniquet hemostasis [12]	Use a hose to deflate blood vessels to stop bleeding.	Firm and reliable.	Wrapping too much will lead to skin damage, and too long will promote tissue ischemia and necrosis.
Pneumatic hemostasis [13]	Filling the tourniquet with gas through a high-efficiency air pump can compress the limbs and block the blood flow.	The operation field of vision is clear to avoid minor structural damage.	It will cause paralysis, shock, pain, skin injury, tissue ischemia, and other adverse reactions.
Single ligation hemostasis [14]	Hemostatic forceps were used to stop bleeding. The ligated tissue was completely sheathed by a ligation line and then ligated.	It can accurately stop bleeding by single ligation of blood vessels.	Postoperative bleeding may occur due to inaccurate ligation or falling off, and too tight will cut blood vessels.
Suture ligation hemostasis [15]	Penetrating ligature hemostasis.	Prevent the ligature wire from falling off.	The suture takes a long time and consumes a lot of sutures, which may lead to uneven tissue alignment.
Electrocoagulation hemostasis [16]	The coagulation current of the probe generates high heat, which promotes the edema of the tissue around the trauma, and the compressed vascular cavity becomes smaller or blocked, forming thrombosis and hemostasis.	Simple, safe, and economical.	Too long will cause the wound to become larger and deeper, resulting in bleeding again.
Ultrasonic scalpel hemostasis [17]	Through the ultrasonic system in the handle, the kinetic energy on the cutter rod is amplified to cut the tissue. After the tissue in contact with the cutter head absorbs the ultrasonic energy, the protein hydrogen bond breaks, and then solidifies, denatures, and cuts into one.	The utility model has the advantages of a wide application range, a clear field of vision during operation, fast cutting, and small cutting tissue damage.	Slow operation, high price, and limited cutting range.
Laser hemostasis [18]	The use of laser coagulation hemostasis, the use of heat energy to evaporate the water in the cells, promotes the degeneration and contraction of vascular wall collagen, and forms small vascular thrombosis.	It has little damage to surrounding tissues, is effective in hemostasis of capillaries and arterioles, and sterilizes at the same time.	It produces toxic smoke and is easy to adhere to after operation.
Microwave knife Hemostasis [19]	The radiation of the microwave knife head generates heat energy, and the tissue absorbing heat energy will solidify and stop bleeding after resection.	The hemostatic effect is obvious; it is not easy to produce a burning taste, the wound heals quickly, and the postoperative bleeding is less.	Only coagulate the blood vessels within 3 mm and temporarily block the blood vessels.
Radio frequency knife hemostasis [20]	Through RF energy, a small plasma electric field is formed in the electrolyte. After the acceleration energy is sufficient, the energy is transmitted to the tissue to destroy the protein ion bond and coagulate.	The heat effect is small, the damage is small, and the saline drops out at the same time as hemostasis.	It can only solidify blood vessels less than 2 mm. A large amount of normal saline is required in the liquid environment, which makes the operation inconvenient.
Argon knife hemostasis [15]	Through the ionized gas conduction of high-frequency current to the tissue, the thermal effect can play a good therapeutic effect.	The gas is stable, the operation does not produce smoke, the fabric damage is small, the continuous solidification is small, and the thermal effect is small. It can form dense eschar, and the hemostatic effect is better.	It can only coagulate blood vessels < 2 mm, which may increase pneumoperitoneum pressure and promote gas embolism and vascular gas embolism.
Ablation hemostasis [21]	Using the thermal tissue effect to dehydrate the tissue and further lead to protein degeneration, coagulation, and necrosis, electrocoagulation and electro-resection of the tissue are carried out in surgery, so as to stop bleeding and cutting.	Avoid accidental tissue injury, reduce tissue adhesion, reduce tissue injury and scar, and shorten the postoperative healing time. It is not easy to produce harmful smoke and clear vision during the operation.	The higher the tissue temperature, the deeper the damage and the worse the safety.

**Table 2:** Commonly used hemostatic materials in medical first aid.

Type	Hemostatic Materials	Advantage	Disadvantage
Polysaccharide-based Hemostatic Hydrogels [30]	Chitosan	Good biocompatibility and degradability, antibacterial and healing-promoting ability, and excellent hemostatic and adhesive properties.	It can be used in patients with coagulopathy. But hemostasis in the wound of extensive bleeding is not very satisfactory.
	Hyaluronic Acid	The ability of rapid hemostasis, accelerating wound healing and preventing infection, is similar to that of fibrin glue.	Poor mechanical properties.
	Alginate	Can accelerate platelet aggregation to accelerate hemostasis, and has good adhesion.	It is suitable for filling the wound, especially the deep and wide surgical cavity, after endoscopic surgery.
	Cellulose	Low cost and excellent mechanical properties.	It is suitable for packaging, application, stuffing, and other operations hemostasis for capillary arterioles and venous bleeding, but it is not suitable for the treatment of peripheral nerve-rich wounds and irregular lacerations.
Protein-Based Hemostatic Hydrogels [30]	Gelatin	Effective control of small blood vessel bleeding, absorption by the body within 4–6 weeks, neutral, can be used with a physiological hemostatic agent.	Water swelling may compress nerves; Use around the site of arterial bleeding may cause displacement of sponges; Use in vascular lacunae may cause embolism.
	Silk	Has unique physical and chemical properties, good mechanical strength, and certain hemostatic abilities.	SF has drawbacks such as brittleness, easy fragmentation, and difficulty in generating a uniform thickness. Further studies are warranted to create a new array of SF-based hemostatic agents.
	Elastin	Components of the extracellular matrix in the vasculature, skin, and lung.	It is insoluble and has poor structural stability.
Inorganic hemostatic agent [31]	Zeolites	Contains inert mineral particles. When it is spread on a wound, the inert particles in it absorb water and clot blood factors.	It cannot achieve rapid hemostasis, and is less effective in arterial bleeding and coagulopathic patients.
	Kaolin	An aluminosilicate clay that activates hemostasis mechanisms.	It cannot achieve rapid hemostasis, and is less effective in arterial bleeding and coagulopathic patients.
Biologically active agents [31]	Fibrin	Fibrin adhesive accelerates the clot tissue in the application area, achieving stable and rapid hemostasis without mixing or other preparation.	The price is high, and there is a risk of disease transmission.
	Thrombin	Local thrombin activates platelets and constrains blood vessels; simple to use and quick to take effect.	The use of animal thrombin may cause an immune response and increase the likelihood of blood clots forming.
	Tranexamic acid	A synthetic lysine analog that acts by blocking the lysine binding site on plasminogen and preventing its activation.	
	Recombinant factor VIIa (rFVIIa)	Significantly reduced transfusion volumes.	Resulting in reduced utilization of blood products in patients with coagulation diseases, high cost, potentially harmful effects, and difficult storage.

## Data Availability

Data sharing is not applicable. No new data were created or analyzed in this study. Data sharing is not applicable to this article.
